# Pancreatic Cancer Epidemiology, Detection, and Management

**DOI:** 10.1155/2016/8962321

**Published:** 2016-01-28

**Authors:** Qiubo Zhang, Linjuan Zeng, Yinting Chen, Guoda Lian, Chenchen Qian, Shaojie Chen, Jiajia Li, Kaihong Huang

**Affiliations:** ^1^Department of Gastroenterology, Lihuili Hospital of Ningbo Medical Center, 57 Xingning Road, Ningbo 315040, China; ^2^Department of Oncology, The Fifth Affiliated Hospital of Sun Yat-Sen University, 52 Meihua East Road, Zhuhai 519000, China; ^3^Department of Gastroenterology, Sun Yat-Sen Memorial Hospital of Sun Yat-Sen University, 107 Yanjiang West Road, Guangzhou 510000, China

## Abstract

PC (pancreatic cancer) is the fourth most common cause of death due to cancer worldwide. The incidence and mortality rates have been increasing year by year worldwide, and this review has analyzed the most recent incidence and mortality data for pancreatic cancer occurrence in China. Several possible risk factors have been discussed here, involving known established risk factors and novel possible risk factors. The development of this cancer is a stepwise progression through intraepithelial neoplasia to carcinoma. Though early and accurate diagnosis is promising based on a combination of recent techniques including tumor markers and imaging modalities, lacking early clinical symptoms makes the diagnosis late. Correct staging is critical because treatment is generally based on this parameter. Treatment options have improved throughout the last decades. However, surgical excision remains the primary therapy and efficacy of conventional chemoradiotherapy for PC is limited. Recently, some novel new therapies have been developed and will be applied in clinics soon. This review will provide an overview of pancreatic cancer, including an understanding of the developments and controversies.

## 1. Epidemiology

Pancreatic cancer (PC), in spite of arising as a thirteenth cancer worldwide, is the fourth most common cause of death due to cancer [1]. The incidence and mortality rates of PC have been increasing year by year worldwide. In 2015, there will be 367,411 new cases and 359,335 deaths from it globally [2]. PC causes about 4.0% of all cancer deaths. In addition, it is an aggressive type of cancer and 80% of patients have locally advanced or metastatic PC at the time of diagnosis. The median survival time for these patients is 4 months and that with metastatic disease is only 2 to 3 months. Sadly, the overall survival rate for patients with PC has not improved over the past two decades.

**Figure 1:**
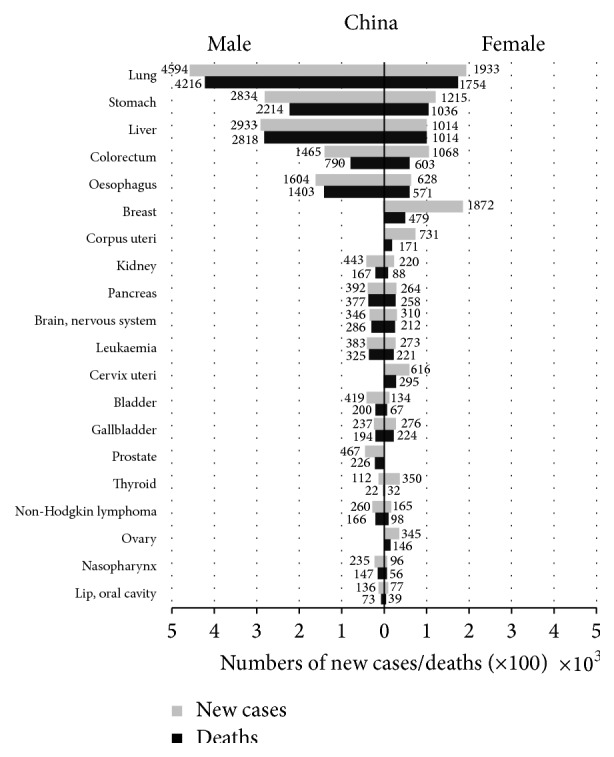
Twenty leading cancer types for the new cases and deaths by sex, China, 2012. GLOBOCAN 2012 (IARC) (13.7.2014) [2].

There is also a continuous increase in PC incidence and mortality in China. According to the recent statistics, it is the seventh most common cancer diagnosis in men and the fourteenth in women and the sixth leading cause of cancer deaths in men and eighth in women.  Figure 1 [2] showed that 65,600 new cases of PC (39,200 men, 26,400 women) and 63,500 deaths (26,400 men, 25,800 women) occurred in 2012. And the rates of incidence and mortality from PC were slightly higher in men than in women.

**Figure 2:**
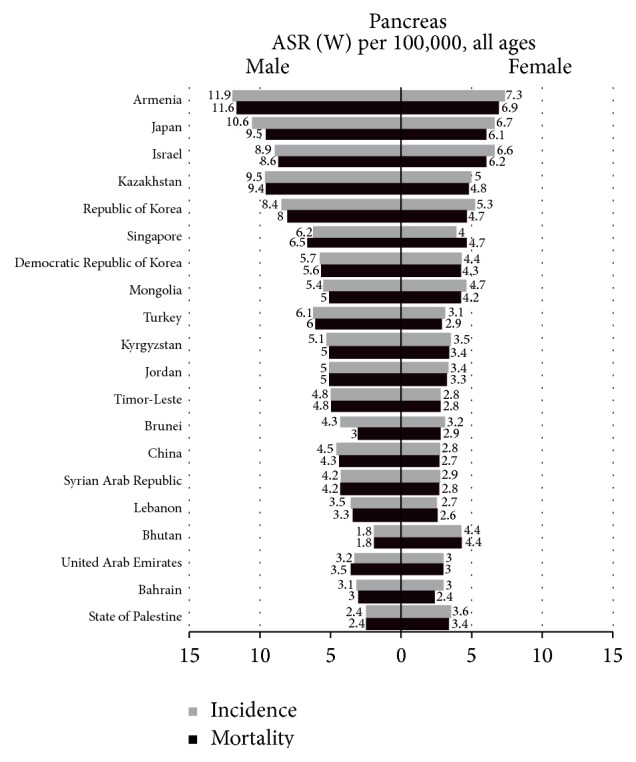
Age-standardized incidence and mortality rates for pancreatic cancer in males and females, China, 2012, from the International Agency for Research on Cancer. GLOBOCAN 2012 (IARC) (13.7.2014) [2].

Figure 2 [2] has shown geographical variations in PC. The age-standardized incidence and mortality rates were calculated with the Asian model population of 2012. ASRs for PC were relatively low in the southern part of Asia, such as State of Palestine, Bhutan, and China. In China, the age-standardized incidence and mortality rates for men were 4.5 and 4.3 per 100,000, while for women they were 2.8 and 2.7. The northern part of Asia showed a considerably higher age-standardized rate (ASR) than countries located in the southern part, for both incidence and mortality. The highest ASR was observed in Armenia, followed by Japan, and Kazakhstan. Among both men and women, ASRs were 2-3 times higher in the north part of Asia. An increasing gradient from the south to the north may suggest a protective factor for PC, which is vitamin D. The serum level of vitamin D among populations in the countries far from the equator with insufficient UV solar radiation is relatively poor. Another possible reason for this difference is the level of economic development. The accuracy of diagnosis for PC is higher in more developed countries.

## 2. Risk Factors

However, because of its relatively low incidence, PC screening in the general population is less effective. As a result, it is urgent to explore the risk factors for PC and to identify the high-risk group. The possible risk factors for PC include gender, age, smoking, alcohol abuse, obesity, physical activities, diabetes, chronic pancreatitis, vitamin D, genetic alterations, dietary, and reproductive factors.

For China, PC incidence is about 48% more common among men than women, as shown in Figure 1. Estrogen and lifestyle habits such as smoking, alcohol abuse may be responsible for the higher morbidity of PC among men than women.

The incidence of PC increases with age, with a slow increase before the age of 50. The median age at diagnosis is 71 years in the United States and 72 years in England. An epidemiological study of China in 2012 showed that 6572,700 had PC diagnosed and about 538,900 (0.8%) had a diagnosis made before the age of 50 [2].

Studies have consistently confirmed that smoking can increase the risk of PC and one-quarter of PC risk might be attributable to smoking. It is associated with 6-fold increase in the risk of PC. There are greater than 60 chemicals identified as prospective carcinogens in cigarette smoke. Of these components swiftly absorbed in the upper aerodigestive tract, nicotine is the major one, which predisposes to PC through causing genetic mutations in pancreatic cells. A recent study revealed that, through Src pathway, the ligation of nicotine and *α*7 nicotinic acetylcholine receptor (nAChR) stimulated metastasis and chemoresistance in PC [4].

Epidemiological evidence suggests that alcohol-abusing group have a higher PC incidence and mortality than nondrinkers. First, chronic pancreatitis, as a known risk factor for PC, is associated with heavy alcohol consumption. Acetaldehyde and fatty acid ethyl esters (FAEEs) are produced in the human body during the metabolism of alcohol and induce pancreatitis-like injury. Second, acetaldehyde has been proved as an organic chemical playing a significant role in carcinogenesis. But the exact mechanism linking alcohol consumption and PC has not been completely defined.

Dietary habits, particularly high-fat diets, resulted in a significant increase of cholecystokinin (CCK). High release of CCK was frequently associated with the development of intravascular tumor emboli, which was correlated with increased vascular endothelial growth factor-A (VEGF-A) [5]. Besides, people who consume a diet high in animal fat are at higher risk for diabetes. A link between diabetes and PC survival has also been suggested, but it remains inconsistent [6]. On one hand, it has been found that patients with long-term diabetes have a 1.5-fold to 2.0-fold increase in the risk of PC; on the other hand, the mean age of developing PC in these patients was significantly older than new-onset ones [7]. Diabetes may even be considered to be a consequence of PC. So, the American Chemical Society recommends that high vegetables and fruits intake might play a role in PC prevention. The role of other dietary habits, such as coffee consumption, drinking tea, remains controversial. For Chinese people, drinking tea is their favorite. Studies in different animals have suggested the effects of tea on tumor formation and growth. In a meta-analysis of studies, it was calculated that consumption of tea may reduce the risk of PC, particularly among Chinese populations and the age group older than 60 years of age [8].

Chronic pancreatitis is a clearly identified and strong risk factor for PC which is up to 20 times greater than the general population [9]. Chronic pancreatitis is a long-term inflammation of pancreas. During the course of inflammation, a variety of pro- and anti-inflammatory mediators (e.g., various cytokines, reactive oxygen species, and cyclooxygenase-2) released from the pancreas promote genomic damage and cellular proliferation and eventually lead to pancreatic malignancy. Tumor-associated macrophages (TAMs), a major inflammatory infiltrate, might link inflammation with cancer. In previous studies, high expression level of TAMs was detected and it might be associated with carcinogenesis, metastasis, and prognosis of PC.

Reproductive factors may be aetiologically associated with PC through estrogen exposure. Several studies, both in vivo and in vitro, have demonstrated that estrogen may lower women's risk of PC. A 100-fold increase in circulating plasma level of estrogen is observed during pregnancy [10]. Women with the higher parity have longer term exposure to high estrogen. And high expression of steroid hormone receptors is frequently found in both benign and malignant neoplasm of pancreas. That is why long-term exposure to estrogen at high concentrations would inhibit the growth of PC, which has been shown in transplanted PC of rodent models. In addition, we know that insulin-like growth factors (IGFs) play a role in PC development, particularly in promoting cellular proliferation and inhibiting apoptosis. A research showed that circulating insulin-like growth factors (IGFs) concentrations in women who had given birth 4 or more times were significantly lower than nulliparous women.

Recently, several genetic susceptibility loci of PC, which account for only 4% of all PC, have been frequently studied in relation to PC risk [11]. For example,* BRCA2*,* PALB2*,* CDKN2a*, and* ATM* germline mutations were carried by 10% to 15% families with familial pancreatic cancer (FPC). In the first stage of pancreatic adenocarcinoma (PDAC), the most common PC,* KRAS* mutations, are particularly frequent. Then, aberrations in* P53*,* STAT3*,* SMAD4,* and* ARF/INK4* are involved in the development of PDAC. Insulin-like growth factor-1 receptor (IGF-1R) is involved in cancer cell metabolism, proliferation, differentiation, apoptosis, and carcinogenesis and chemoresistance [12]. Overexpression of IGF-1R in PC cells has been reported. In recent study, silencing IGF-1R could negatively regulate PC growth and metastasis via suppressing key signaling pathways such as PI3K/AKT, MAPK, JAK/STAT, and EMT. Moreover, dysregulated genes involved in pathways, such as Sonic Hedgehog (Shh), Wnt, Notch, and transforming growth factor *β* (TGF-*β*) signaling, have revealed association with pancreatic tumor formation. Families with hereditary pancreatic cancer syndromes are considered at high risk. There are six certain hereditary conditions [13], such as multiple endocrine neoplasia type 1 (MEN1) syndrome, hereditary nonpolyposis colon cancer (Lynch syndrome), von Hippel-Lindau syndrome, Peutz-Jeghers syndrome, hereditary breast/ovarian cancer, and familial atypical multiple mole melanoma (FAMMM) syndrome.

## 3. Pathophysiology

The development of PC is a stepwise progression involving activation of oncogenes, inactivation of tumor suppressor genes, and deregulation of the cell cycle. There are three morphologic forms of noninvasive pancreatic neoplasia differing in biological and clinical behavior. These are (1) intraductal papillary mucinous neoplasm (IPMN) which is composed of mucin-producing neoplastic cells growing in the main pancreatic duct or in one of its major branches, (2) mucinous cystic neoplasm (MCN), as another mucinous cystic neoplasm, which does not connect to the native pancreatic ductal system and can be separated into three categories (benign, borderline, and malignant), and (3) pancreatic intraepithelial neoplasia (PanIN) which is the most common precursor to PC in human, proposed by Klimstra and Longnecker as a “gold standard” for describing the noninvasive lesions.

**Figure 3:**
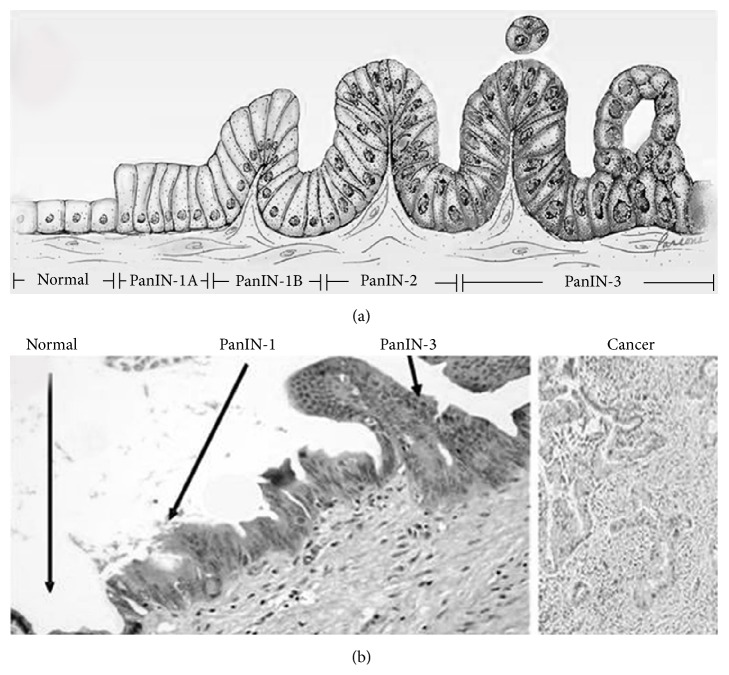
Histological progression from normal pancreatic cells to pancreatic intraepithelial neoplasia [3]. (a) Model for histological progression from normal pancreatic cells to pancreatic intraepithelial neoplasia (PanIN). (b) Micrograph of normal pancreas, pancreatic intraepithelial neoplasia (PanIN), and pancreatic cancer (PC) [from Wikipedia]. Gradual transition from PanIN-1 to PanIN-3 was observed in a single duct. Haematoxylin and eosin stain.

PanINs are microscopic lesions initiating in small-caliber pancreatic ducts (<5 mm diameter) and may be classified into four consecutive stages accompanied by cumulative genetic alterations, as shown in Figures 3(a) and 3(b) [3]. Low-grade PanIN lesions (PanIN-1A/ PanIN-B) are flat or papillary epithelial lesions, which are characterized by epithelial cells with columnar shape and basally oriented uniform nuclei. As indicated above, activating KRAS mutations occur first (in PanIN-1 lesions).

As indicated above, TAMs might link inflammation with PC and play an important role in tumor growth and metastasis [14, 15]. In the tumor microenvironment, TAMs are mainly polarized towards M2 phenotype macrophages. In Japan, several studies have shown that high number of infiltrating M2-polarized macrophages in tumor tissue is related to a poor prognosis in PDAC patients [16–18]. In our previous study, we also found that TAMs infiltration had a strong association with the incidence of lymph node metastasis [19]. In 2002, it was reported that TAMs expressed vascular endothelial growth factor- (VEGF-) C and impacted tumor lymphangiogenesis in the peritumoral inflammatory microenvironment [20]. The results indicate that TAMs may have the ability to release cytokines and chemokines to affect tumor cell microenvironment, which enable lymph node metastasis. Additionally, 41 (58.6%) patients with PDAC in our previous study suffered from abdominal pain, and it was significantly associated with a higher level of infiltrating TAMs. These findings indicate that TAMs may involve the procedure of PC neural invasion. In the future, the clear molecule mechanism of TAMs in the PC tumor microenvironment requires further investigation.

The oncogenic* KRAS* ^*G12D*^ is associated with invasive adenocarcinoma, through regulating division, differentiation, and apoptosis of pancreatic cells. The altered guanosine triphosphatase (GTPase) shows an increased activity of Ras-GTP, which stimulates downstream effector, namely, AKT. Activation of phosphatidylinositol 3 kinase (PI3K)/AKT pathway increases cell proliferation, survival, and protein synthesis in PC. In addition to its critical role in tumor initiation,* KRAS* is essential for the maintenance of PC. Compared to PanIN-1, PanIN-2 lesions are mostly papillary with higher nuclear atypia, including loss of nuclear polarity, nuclear crowding, enlarged nuclei, nuclear hyperchromasia, and nuclear pseudostratification. The inactivation of* p16INK4A/CDKN2A* gene occurs usually in PanIN-2. This tumor suppressor gene encodes protein p16, which binds to cyclin-dependent kinase 4/6 (Cdk4/6) and arrests cell cycle in G1 phase. Loss of the cycle-dependent kinase inhibitor, protein p16, arrests apoptosis. In PanIN-3 lesions, small clusters of epithelial cells with nuclear pleomorphism and high mitotic rate bud off into lumen. The lesion is a noninvasive form, known as “carcinoma in situ” of pancreatitis ductal adenocarcinoma (PDAC). In the progression from PanIN-3 to adenocarcinoma, accumulation of genetic alterations is detected, such as mutation in* TP53*,* DPC4*, and* BRCA2*. Maintenance of the G2/M arrest is dependent on a tumor suppressor, TP53. Another tumor suppressor gene,* DPC4*, was not found to be inactivated in PanIN-1/2. Inactivation of* DPC4* induces disruption of TGF-*β* pathway, then leading to subsequent cell growth, differentiation, and oncogenesis. Compared to* TP53* and* DPC4*, loss of* BRCA2* occurs even later.* BRCA2*-mediated DNA repair is the most critical in the maintenance of genomic integrity. Mutations in* BRCA2* cause an increased risk for PC. Though infiltrating adenocarcinomas are believed to develop from adjacent PanINs, the clinical significance of PanINs in the transection margin remains undefined.

Most pancreatic tumors are exocrine tumors, including ductal adenocarcinoma, acinar cell carcinoma, cystadenocarcinoma, adenosquamous carcinoma, signet ring cell carcinoma, hepatoid carcinoma, colloid carcinoma, undifferentiated carcinoma, pancreatoblastoma, and pancreatic mucinous cystic neoplasm. And the most common form is ductal adenocarcinoma characterized by moderately to poorly differentiated glandular structures, comprising 80% to 90% of all pancreatic tumors. Pancreatoblastoma mostly occurs in childhood and it has a poor prognosis when it occurs in adult. Pancreatic mucinous cystic neoplasms range from totally benign to malignant, which can be diagnosed by EUS with cyst fluid analysis. By contrast, endocrine pancreatic tumors, the so-called neuroendocrine tumors of pancreas (PNET), are rare and they account for only 1-2% of all pancreatic tumors. Clinical manifestations of PNETs are varied based on the degree of differentiation and functionality. For many years, clinicians considered that these tumors displayed benign behavior with good prognosis. However, evidence has suggested that all PNETs larger than 0.5 cm were malignant.

Usually, PC is likely to metastasize early and rapidly, which is the primary cause of death. It first spreads to regional lymph nodes, followed by the liver and the peritoneal cavity. The prevalence of neural invasion is high, which is considered to be associated with abdominal pain. Metastasis to the lungs, bones, and brain is unusual. It is rare that PC metastasizes to the skin, which is called cutaneous metastasis, commonly to the umbilicus. But there have been a few number of cases of nonumbilical cutaneous metastases reported. In 2015, a case of 58-year-old PC patient with muscular metastasis was reported in France [21]. However, the mechanism of pancreatic tumor metastasis remains unknown. There are various studies on the mechanism of metastasis. As shown previously by Poomy P et al., high expression of amyloid precursor-like protein 2 (APLP2) is positive correlative to highly metastatic PC cells. Proliferator-activated receptor-*γ* (PPAP-*γ*), a nuclear receptor, is well accepted as a transcription factor in metastasis of PC. And the present studies aimed to investigate whether ligands of PPAP-*γ*, such as thiazolidinediones (TADs), inhibit metastasis of PC cells [22].

## 4. Diagnosis and Staging

Early and accurate diagnosis of PC, which often can be challenging, is important because it helps doctors choose the effective and timely treatment option for patients. It is usually based on a combination of imaging techniques such as computer tomography (CT) and endoscopic ultrasonography (EUS), tumor markers such as carbohydrate antigen 19-9 (CA19-9), clinical presentations, and the “gold standard” diagnosis-biopsy.

### 4.1. Clinical Presentation

To date, lack of symptoms is the main cause of PC late diagnosis and therapy. The appearance of clinical presentations usually indicates an advanced stage and the most frequent presentations are progressive weight loss, anorexia, abdominal pain, and jaundice. These symptoms of PC are nonspecific and varied in different parts of pancreas. The tumor in the head of the pancreas (75%) produces symptoms such as weight loss, painless jaundice, nausea, and vomiting. The mass of pancreatic head causes blockage of the common bile duct, which results in jaundice, dark urine, light stool color, and itching. Weight loss may be related to malabsorption of nutrients due to PC. Nausea, vomiting, and poor appetite, due to cancer-related gastric outlet (duodenum) obstruction, may also contribute to weight loss. If cancer is located at the body/tail of the pancreas, patients usually present with abdominal pain that radiates to the sides or the back. Previous reports showed that inflammatory and immune cells were associated with both the pain intensity and the extent of perineural invasion (PNI). And PNI is also involved in pain generation. Due to PC producing blood clotting chemicals, thrombus forms automatically in the portal blood vessels, the deep veins of the extremities, or the superficial veins on the body, which is known as Trousseau syndrome. In comparison to patients with other types of digestive cancer, patients in advanced stage of PC will experience more anxiety and depression. Earlier studies indicate that proinflammatory cytokines may be responsible for cancer-related depression. And increased levels of several cytokines including interleukin-6 (IL-6), interleukin-8 (IL-18), and TNF-*α* in patients with PC have been found. These cytokines may correlate with the regulation of hypothalamic-pituitary-adrenal (HPA) axis and corticotropin-releasing factor (CRF). However, this hypothesis remains unclear. Other common symptoms include fatigue, diarrhea, and heartburn.

### 4.2. Tumor Markers

In the screening of asymptomatic patients with PC, the clinical role of serologic markers, which includes CA19-9, carcinoembryonic antigen (CEA), osteopontin (OPN), macrophage inhibitory cytokine 1 (MIC-1), and S100A6, has been limited.

CA19-9 is an isolated Lewis antigen of the tumor-associated protein mucin 1 (MUC1). It can be helpful in the assessment of response to chemotherapy, in the early detection of tumor recurrence, and even in the predicting of the prognosis. The role of CA19-9 in PC diagnosis is inconclusive, though it is the most useful and routinely adopted, because highly elevated serum level of CA19-9 has been found in many other gastrointestinal tumors and ovarian cancer, as well as nonmalignant diseases. Chronic inflammation or acute injury may induce CA19-9 synthesis through pathologic fibrosis, which has been approved by immunohistochemical analysis for CA19-9 in hepatic inflammatory areas and bile ductule cells. That may be the reason why CA19-9 is elevated in chronic hepatitis and nonmalignant objective jaundice. Besides, CA19-9 level cannot be elevated in 10% of Caucasians even with large pancreatic tumor because they are Lewis-negative.

CEA, another biological marker for prognosis of PC, is a glycoprotein. A rising CEA level is associated with adenocarcinoma, including colon cancer, breast cancer, and stomach cancer. The sensitivity and specificity of CEA in PC were 83.78 and 69.44%, respectively. The level of CEA has significant correlation with tumor size, tumor differentiation, and lymphatic and liver metastasis.

Serum OPN is one of the most recent biomarkers that have shown potential clinical applicability for PC. It is a highly phosphorylated sialoprotein discovered in 1986 in osteoblasts. Proinflammatory cytokines (e.g., TNF-*α*, IL-1*β*, and angiotensin II) upregulate the expression of OPN. Moreover, elevated levels of OPN were also found in a variety of cancers, including lung cancer, stomach cancer, and PC. It may promote cancer metastasis through the ligand-receptor interaction with the CD44 receptor family. A meta-analysis revealed that OPN was a serum diagnostic biomarker for the early-stage PC [23].

A previous study indicated that MIC-1 was a potential diagnostic biomarker in early diagnosis and postoperative monitoring for PC [24]. As a member of the TNF-*β* superfamily, MIC-1 is weakly expressed under normal conditions, but it is markedly upregulated in inflammatory diseases as well as cancers. Compared to CA19-9, MIC-1 seems to have better sensitivity; however, it has lower specificity in differentiating pancreatitis from PC.

A number of proteins in the S100 family have been found to be related to PC progression and metastasis. S100A6 is a member of this family and PC patients with high level expression of S100A6 have poor outcome. It is significantly elevated in intraductal papillary mucinous neoplasms (IPMN), in pancreatic tumors, and even in PanIN lesions. S100A6 protein may influence the invasion of PC, but it is not yet clear what the precise mechanism is.

In addition, several fecal markers have been studied, such as methylated bone morphogenetic protein 3 (mBMP3) and Adnab-9. In stools from PC patients, significantly higher mBMP3 was found when compared to stools from the controls. And* BMP3* is recognized as a tumor suppressor. We therefore hypothesized that aberrant* BMP3* promoter methylation PC led to the development of PC. The presence of Adnab-9 in stools has been shown to be associated with PC precursor lesions. As a fecal biomarker, Adnab-9 has a sensitivity of 80% and a specificity of 87% for the detection of PC [25]. In the future, effective tumor markers can be used to aid in the diagnosis of the presymptomatic PC, treatment assessment, and then monitoring for disease recurrence.

### 4.3. Imaging

Over the years, imaging techniques, such as transabdominal ultrasound (US), CT, magnetic resonance imaging (MRI), positron emission tomography- (PET-) CT, endoscopic retrograde cholangiopancreatography (ERCP), and EUS, play a vital role in PC detection and staging.

Although in small pancreatic lesions (less than 3 cm) detection with fairly low sensitivity (67%) and specificity (40%) for PC, US is the most widely used image technique. Because it is inexpensive, safe, and painless, US is strongly recommended as the initial screening tool for PC.

As one of the most convenient imaging tools, the new methods in the development of CT scans, including multidetectors, intravenous contrast, curved planner reformations, CT angiography, and some postprocessing techniques, have shown promise in detection and staging of PC. Helical CT may detect masses larger than 2 cm with a sensitivity of 78–100%. CT imaging postprocessing techniques have greatly enhanced its ability in preoperative TNM staging of PC. Planar reformatted images and curved reformatting are now being used to detect PC location and its relationship to adjacent structures, such as the pancreatic duct, common bile duct, and blood vessels. Maximum intensity projection (MIP) and volume rendering can identify narrowing or irregularity of vessels due to tumor encasement. In spite of some disadvantages of CT such as considerable radiation exposure and potential for contrast-induced nephropathy, it is a comprehensive primary imaging modality for PC diagnosis and staging.

MRI can help to clearly define pancreatic mass without abnormal CT findings. It is superior to CT in the detection of small pancreatic tumors, hypertrophied pancreatic head, isoattenuating pancreatic cancer, and focal fatty infiltration of parenchyma. Furthermore, magnetic resonance cholangiopancreatography (MRCP), which can delineate the pancreatic ductal system noninvasively, is currently used as an accurate diagnostic tool for patients with suspected biliopancreatic disease.

PET-CT with fluorine-18 fluorodeoxyglucose (^18^F-FDG) is a combination of PET and high-end multi-detector-row CT, being widely used for diagnosis, staging, and monitoring cancer following treatment, such as PC. ^18^F-FDG PET/CT can detect the metabolic activity in PC and evaluate pancreatic tumor response to radiotherapy.

Another important technique in the diagnosis of PC is ERCP, which combines upper gastrointestinal (GI) endoscopy and fluoroscopy. It provides direct visualization of pancreatic and bile duct system with morphologic alterations, like stenosis and dilation. The sensitivity of ERCP with respect to diagnosing pancreatic cancer was 70% and its specificity was 94% [26]. Moreover, during ERCP, we can collect the pancreatic juice and cells for pathological examination. But it is invasive and may cause some related complications, such as bleeding, perforation, and pancreatitis. The information of tumor size provided by ERCP is limited and metastasis cannot be assessed.

Compared to ERCP-based brush cytology, the accuracy rates of EUS-guided fine needle aspiration (FNA) of pancreatobiliary tumors are higher (over 80%) [27]. Furthermore, EUS is critical for preoperative staging of pancreatic head tumor by virtue of determining nearby blood vessels and lymph nodes involvement.

### 4.4. Staging

**Figure 4:**
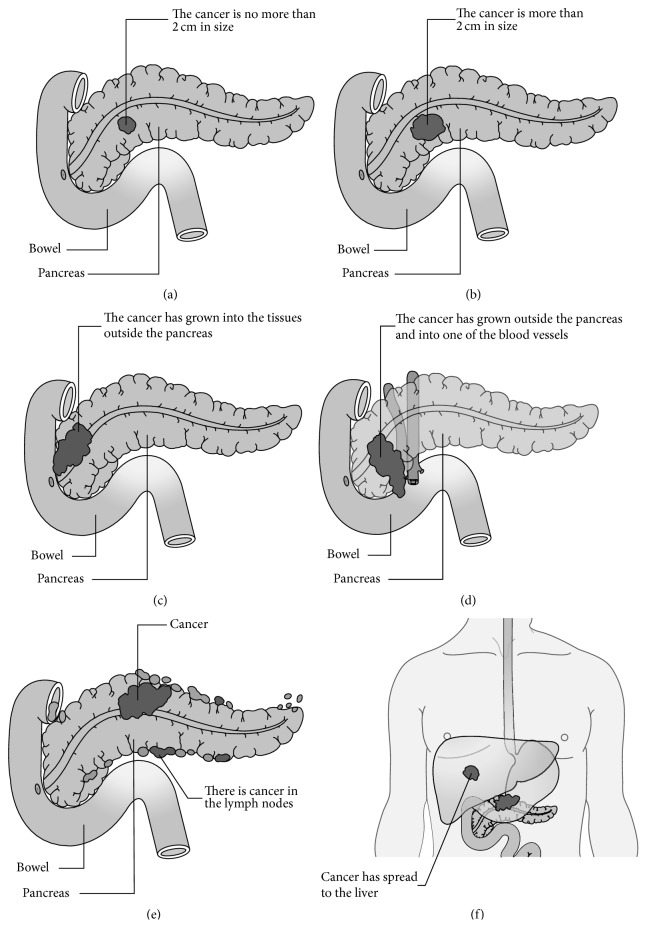
The UICC (Union for International Cancer Control)/AJCC (American Joint Committee on Cancer) Staging System for PC.

Prognosis and treatment depend on the stage of PC at diagnosis. Therefore, correct staging is critical. Staging is principally based on UICC (Union for International Cancer Control) TNM classification for PC (Figure 4 [28], Table 1). According to a simpler practical staging system, patients with PC can be divided into “resectable,” “borderline resectable,” and “unresectable,” which was made at surgical exploration in the past. As modern imaging techniques are being developed, preoperative staging is now becoming available. Patients, who thought to have resectable cancers, include those with stage I and stage II cancers. However, local treatments such as radiation is considered as an option for stage III PC and chemotherapy is used as the only treatment for patients with stage IV PC. In recent years, a general agreement has been reached that patients without distant metastases but with blood vessel involvement (>180° of superior mesenteric vein/portal vein, <180° of superior mesenteric artery, occlusion or deformity of a short segment) are considered as the “borderline resectable” group. Borderline patients may benefit from the survival from neoadjuvant therapy.

## 5. Management

Despite the improvement of PC diagnostic tests over these years, the rate of diagnosis at an early stage remains low, and so is the survival rate. These days, the efficacy of conventional chemoradiotherapy for PC is limited, and surgery is the best option for these patients.

### 5.1. Surgical Resection

Surgery remains the only possibility for curing of PC, though there are only 20% of patients with operable PC. The selection of an operative procedure for PC is based on factors such as the tumor location, tumor size, and tumor staging.

The classic Whipple procedure (pancreatoduodenectomy), which involves removing the pancreatic head, as well as the curve of the duodenum, the gallbladder, and the common bile duct, is the most common operation for cancers of the head and/or neck of pancreas. In 1898, Alessandro Codivilla performed this procedure firstly on a patient with PC [29]. Unfortunately, this patient died of disseminated recurrence 24 days after surgery. Since an American doctor named Allen Oldfather Whipple devised the perfect version in 1935, it is called the Whipple procedure, which is performed on patients with pancreatic head cancer and periampullary cancer.

When cancer involves the body and tail, distal/subtotal pancreatectomy is suggested. About 35% of the patients with body/tail PC were observed at the time of surgery, finding that the tumors have spread to surrounding tissues. In such cases, extended resection should be advisable. For patients with locally advanced pancreatic cancer (LAPC), multivisceral resection is technically feasible. Based on recent publications, perioperative mortality (3%) and morbidity (35%) did not differ between two groups of patients who underwent standard resection or multivisceral resection [30, 31]. Although laparoscopy for PC treatment is controversial, laparoscopy in diagnosis and staging of PC is known to be critical, safe, and reliable. Refinements in surgical techniques will reduce perioperative morbidity and improve the outcomes.

### 5.2. Chemotherapy

For unresectable PC, chemotherapy is being extensively used, such as GEM/erlotinib, FOLFIRINOX, GEM/NAB-paclitaxel, GEM/capecitabine, and capecitabine/oxaliplatin (XELOX). However, PC is characterized by a dense desmoplastic reaction which promotes resistance to chemotherapy [32].

As the key drug for chemotherapy of unresectable PC, gemcitabine (GEM) was first synthesized by Larry Hertel at Eli Lilly during the early 1980s. With the introduction of many new agents, such as 5-fluorouracil (5FU), cisplatin, epirubicin, oxaliplatin, leucovorin, and irinotecan, there have been multiple chemotherapy regimens for PC. In PC cells, epidermal growth factor receptor (EGFR) is overexpressed, and erlotinib is an EGFR-tyrosine kinase inhibitor. As a result, the level of EGFR expression may predict the efficacy of this combined chemotherapy in PC. And then, in 2011, Conroy et al. suggested that FOLFIRINOX, a combination regimen of oxaliplatin, 5FU, leucovorin, and irinotecan, should be used as first-line systemic chemotherapy in patients with advanced PC [33]. Because FOLFIRINOX toxicity is higher than GEM alone, this regimen is considered as an option for younger patients with a good performance status. More recently, the efficacy and safety of another combined chemotherapy of GEM plus NAB-paclitaxel (NAB-P) has been well evaluated in a clinical trial. NAB-P is an albumin nanoparticles, which is water-soluble formulation with less toxicity and a relatively higher local concentration in stromal-rich tumors. GEM sensitivity can be enhanced through inhibiting the primary GEM catabolic enzyme by NAB-P [34]. In addition, capecitabine (CAP) is widely used as an orally administered prodrug that is enzymatically converted to 5FU by thymidine phosphorylase (dThdPase) preferentially located in tumors. So, CAP is much safer, more effective, and convenient than 5FU. Besides CAP, oxaliplatin is active as primary therapy for advanced PC. However, the combination of CAP plus oxaliplatin (XELOX) is just used as second-line chemotherapy because of limited experience. In spite of limited efficacy in metastasis PC, chemotherapy plays a central role in the adjuvant setting for patients with metastasis PC.

### 5.3. Radiotherapy

For unresectable PC, there is little evidence to support the efficacy of radiotherapy. However, radiotherapy can be used as a palliative treatment option for those unresectable locally advanced tumors. It can kill cancer cells and keep them from growth and recurrence. People will have side effects from radiotherapy, such as fatigue, gastrointestinal symptoms, skin rashes, and toxicity to the surrounding normal tissues. Fortunately, recent innovation in radiotherapy symbolized by intensity-modulated radiotherapy (IMRT) and image-guided radiotherapy (IGRT) provides alternative treatment which is much more effective and tolerable [35]. These technologies allow an increase of the target volume dose while minimizing the dose to the surrounding normal structures. As in a previous study, IGRT and IMRT after preradiation chemotherapy for longer than 9 months improved overall survival and progression-free survival for these PC patients. In addition, induction of radiosensitization by injection of hydrogen peroxide and sodium hyaluronate into the unresectable pancreatic tumor would enhance the efficacy of radiotherapy, without serious complications [36]. Because of highly advanced technologies in radiotherapy, a new precisely targeted radiotherapy, named stereotactic body radiotherapy (SBRT), has been applied to treat PC, which has been successful in the treatment of thoracic tumors and early-stage non-small-cell lung cancer. It can deliver a high dose of radiation accurately while minimizing the dose to the surrounding normal tissues. Further investigation of radiotherapy is needed to improve its efficacy and safety in the treatment of local advanced PC.

### 5.4. Other Treatment Strategies

New promising therapies are urgently needed because only a few patients with PC can benefit from conventional treatments, like chemotherapy or radiotherapy. Gene therapy in PC is not yet applied in clinics, although it has become successful in vitro as well as in vivo. It includes gene replacement, gene modification, and gene blockade. PC gene therapy is mainly based on target genes, such as* p16INK4A/CDKN2A*,* p21CIP1/WAF1*,* p14ARF*,* K-ras*,* LSM1/CaSm*,* HER-2/EerB-2*,* MDR1*,* BCRP*, and* VEGF*. A bacterial cancer vaccine for PC, using a live attenuated* Listeria* strain as vector, is just beginning to reach early-phase clinical trial [37]. Recently, a novel new treatment has been developed, which is called high intensity focused ultrasound (HIFU). The first-in-human clinical trial of high intensity focused ultrasound (HIFU) in advanced PC was performed in China in 2000. Utilizing high intensity focused ultrasound energy, it causes all the targeted PC cells necrosis through heating. The effect of HIFU in ablation is a combination of direct and indirect effects. The direct effect includes thermal ablation on targeted cancer cells, mechanical effects such as acoustic pressure and shear stress. Indirect effect is associated with tumor blood vessel destruction. HIFU is a palliative treatment with less invasive and shorter recovery, which offers a suitable alternative. Another highly potent approach, which has been tested only in studies involving animals, is to enhance cancer cell death through an antiglycolytic agent called 3-bromopyruvate (3-BrPA) [38]. 3-BrPA inhibits enzyme activity of glyceraldehydes-3-phosphate dehydrogenase (GAPDH), one of the key glycolytic enzymes. Julius et al. developed the formulation of 3-BrPA, microencapsulated in a complex with *β*-cyclodextrin (*β*-CD), which limited exposure of 3-BrPA to normal cells. In the future, we wish to develop more and more novel therapeutic strategies, which could prove to be promising for PC patients.

## Figures and Tables

**Table 1:** Staging group for PC.

UICC disease stage	T staging	N staging	M staging
Stage 0	Tis	N0	M0
Stage IA	T1	N0	M0
Stage IB	T2	N0	M0
Stage IIA	T3	N0	M0
Stage IIB	T1–3	N1	M0
Stage III	T4	Any N	M0
Stage IV	Any T	Any N	M1

Tis: carcinoma in situ (the tumor is confined to the top layers of pancreatic duct cells. Very few pancreatic tumors are found at this stage).
